# Leaf Tissue Macronutrient Standards for Northern Highbush Blueberry Grown in Contrasting Environments

**DOI:** 10.3390/plants11233376

**Published:** 2022-12-05

**Authors:** Scott Lukas, Shikha Singh, Lisa Wasko DeVetter, Joan R. Davenport

**Affiliations:** 1 Department of Horticulture, Oregon State University, 2750 SW Campus Way, Corvallis, OR 97331, USA; 2 Department of Horticulture, Northwestern Washington Research and Extension Center, Washington State University, Mount Vernon, WA 98273, USA; 3 Department of Crop and Soil Sciences, Irrigated Agriculture Research and Extension Center, Washington State University, Prosser, WA 99350, USA

**Keywords:** *Vaccinium corymbosum*, nutrient management, tissue testing, fertilization, calcareous soil

## Abstract

Leaf tissue testing is a useful tool for monitoring nutrient requirements in northern highbush blueberry (*Vaccinium corymbosum* L.; abbreviated as “blueberry”) but may require adaptation to specific growing environments. The objective of this study was to evaluate macronutrient concentrations in early-, mid-, and late-season blueberry cultivars grown in two contrasting environments, specifically eastern and western Washington. Climate and soil conditions between these two regions differ tremendously with eastern Washington being more arid with naturally calcareous soils lower in soil organic matter. Sampling was conducted over a 3-year period in commercial fields. Leaf tissue nitrogen (N), phosphorus (P), potassium (K), calcium (Ca), magnesium (Mg) and sulfur (S) concentrations were affected by year (Y), growing region (R), cultivar (C), and Day of Year (DOY) that the samples were collected with many interactions. Leaf nutrient concentrations were higher, on average, in western than eastern Washington except for Ca and Mg, indicating sufficiency levels should differ between these regions. Leaf macronutrients generally stabilized between DOY 212–243 (1–31 August), suggesting this period is optimal for tissue sampling. Findings from this study demonstrate the importance of considering regional effects and may be applicable for blueberry cultivated in similar pedo-climactic conditions around the world.

## 1. Introduction

Northern highbush blueberry (*Vaccinium corymbosum* L.; abbreviated “blueberry” hereafter) is a globally important fruit crop valued for its berries in fresh or processed forms. The species is a calcifuge with unique soil and nutrient requirements including low soil pH (4.5–5.5), high soil organic matter (OM) levels (>4%), and low-to-moderate soil fertility [1,2,3,4]. Nutrients below or in excess of crop needs can lead to yield reductions, reduced fruit quality, and economic losses [5,6,7,8,9]. Overfertilization may also contribute to environmental pollution if nutrients leach through the soil profile or are moved off site through other mechanisms as reviewed in [10,11]. These unique soil and nutrient requirements can make nutrient management challenging. A successful blueberry nutrient management program incorporates analyses of leaf nutrients, along with observations of soil pH, soil nutrient concentrations, plant growth, and productivity [1,4,12]. Annual analysis of leaf nutrients allows managers and crop advisors to see trends in nutrient concentrations over time as well as assess whether nutrients are sufficient, deficient, or in excess. This information in turn is used to inform nutrient management programs. However, nutrient standards are needed to enable these evaluations and may require adaptation to specific growing environments that vary in climate and soil conditions.

Washington and Oregon are the leading blueberry producing states in the geographic region known as the Pacific Northwest within the United States of America (USA). In 2021, these two states combined produced 150,000 t of blueberries, representing 49% of USA production, with an economic value of $228 million [13]. Production in the Pacific Northwest is separated geographically and climatologically into two distinct areas by the north–south oriented mountain system called the Cascade Range [14]. This range intercepts the path of the moisture-laden marine air moving in from the Pacific Ocean. The resultant cooling and condensation of this air produces some of the heaviest annual rainfalls in the USA along the western areas of the range, creating the mild and humid maritime climate of western Washington and Oregon. Precipitation amounts decrease rapidly once the mountain crest is crossed, forming a rain shadow on the leeward side producing a semiarid climate in eastern Washington and Oregon [14,15].

The effect of natural precipitation on the soils east and west of the Cascade Range in the USA has created distinctively different soil properties. The interaction of natural precipitation with carbon dioxide in the atmosphere creates a dilute carbonic acid which adds hydrogen ions (H^+^) to the soil [8]. These H^+^ replace cations, including calcium (Ca^2+^), magnesium (Mg^2+^), and potassium (K^+^), which are attracted to or held on the surface of soil particles. Centuries of leaching by winter rainfall have developed the naturally acidic soils (pH 4.8 to 6.2) west of the Cascade Range [8]. By contrast, the limited amount of natural precipitation east of the Cascade Range has slowed the natural soil acidification process, resulting in a soil pH of 7.8 to 8.2 [16].

The factor that determines the rate at which soils acidify is the soil buffering capacity. Buffering capacity is primarily a function of cation exchange capacity (CEC), which is affected by clay composition of soil and OM [16]. Soil with higher clay and OM content have increased CEC and buffering capacity, leading to a decreased soil acidification rate [16]. The impact of OM is not only a factor affecting buffering capacity, but also a prerequisite for understanding the availability and cycling of nutrients such as carbon (C), nitrogen (N), sulfur (S), and phosphorus (P) [17]. Accumulation of OM in the soil is crucial for soil fertility, water retention, and maintaining crop productivity [18]. Soils west of the Cascade Range contain high amounts of OM (>4%) compared to those east of the Cascade Range (<0.7%), indicating soil fertility requirements may differ between these two regions.

To satisfy the soil requirements to successfully produce blueberries, high-pH soils east of the Cascade Range must be artificially acidified [19,20] and frequently are amended with organic materials such as woodchips obtained from regional apple (*Malus domestica* Borkh.) and sweet cherry (*Prunus avium* L.) orchards. Soil acidification by means of elemental sulfur (S°) prior to planting or with acids during the growing season through the irrigation system are common field practices worldwide [21]. Depending on initial pH and buffering capacity of the soil, high amounts of S° may be needed to lower the pH to an optimal level for blueberry [19]. Soil acidification, plus amendments with organic materials, may change the availability (although not chemistry) of some of the naturally occurring soil nutrients [22], as well as nutrients added through growers’ fertilizer management practices. As a result, nutrients may be more or less available depending on the nutrient and soil chemistry within variable production regions, justifying different nutrient management programs to meet plant demand. Bair and Davenport [23] demonstrated that even when soils in the naturally high pH region of eastern Washington have been acidified through management practices, nutrient availability remains similar chemically to that found in natively low pH soils. The high pH values present in soils east of the Cascade Range likely impact plant growth and nutrient requirements, as well as mineralization rates and synchronization of nutrient availability with plant demand [24].

For the most part, leaf tissue nutrient standards have been developed in a single region and then translated across production regions for the crop. Current tissue nutrient standards for blueberry in the USA are based on standards developed in Michigan [25] and modified in the Pacific Northwest from research in western Oregon [1,12]. Published nutrient sufficiency levels provide a good starting point to develop guidelines but may need to be tailored for each region or growing area with unique climactic and soil conditions. Tissue nutrient levels as well as timing of sampling may vary widely throughout production areas and across cultivars with various fruiting seasons. For example, sampling leaves at 2 weeks after harvest is recommended for southern highbush blueberry (*V. corymbosum* L. interspecific hybrids) in the southeastern USA. Ref. [26] but sampling immediately after fruit harvest is not suitable for northern highbush blueberry in Michigan [25] or western Oregon [1,12]. In western Oregon, Strik and Vance [12] found that the best sampling time to determine plant nutrient status in northern highbush blueberry is the period between late-July and mid-August, which is when the concentration of most nutrients in the leaves are relatively stable, regardless of cultivar or fruiting season. It is unclear if the optimal sampling time for leaf tissue should differ east and west of the Cascade Range with the earlier fruiting seasons found in the region.

The overarching goal of this study was to evaluate and establish regional leaf tissue macronutrient sufficiency standards and leaf sampling time guidelines for blueberry grown in two contrasting environments, eastern and western Washington. Specific objectives were to: (1) Evaluate and establish macronutrient sufficiency standards for blueberry grown in eastern and western Washington; and (2) determine the best time for sampling leaves for early, mid-, and late-season northern highbush blueberry cultivars in eastern and western Washington. More broadly, findings from this study could be applicable to other blueberry production regions with similar pedo-climatic zones as the ones considered in our study.

## 2. Results

The concentration of N, P, K, Ca, Mg, and S in the leaves were significantly affected by growing region (R), cultivar (C), and the day of year (DOY) that the samples were collected. Most interactions were also significant except C × DOY for Mg and R × C × DOY for N, P, K, Ca, and Mg (Table 1) in 2015. In 2015, leaves from western Washington showed significantly higher concentrations of N, P, and K than eastern Washington and contrasting trends were observed for Ca and Mg. Similar trends were observed in 2016 and 2017. The cultivars were also compared for the three years separately (Table 1). In 2015, all nutrients were significantly influenced by cultivar except P while in 2016 and 2017, higher N in leaves were observed in mid-season cultivars than early- and late-season cultivars (Table 1). Similar trends were observed in P, Ca, and S. Leaf nutrient concentrations were also significantly influenced by DOY. For N, P, and K, higher concentrations were observed in early DOYs in all the years while for Ca, higher leaf concentrations were observed in later DOYs for all the three years (Table 1).

**Figure 1:**
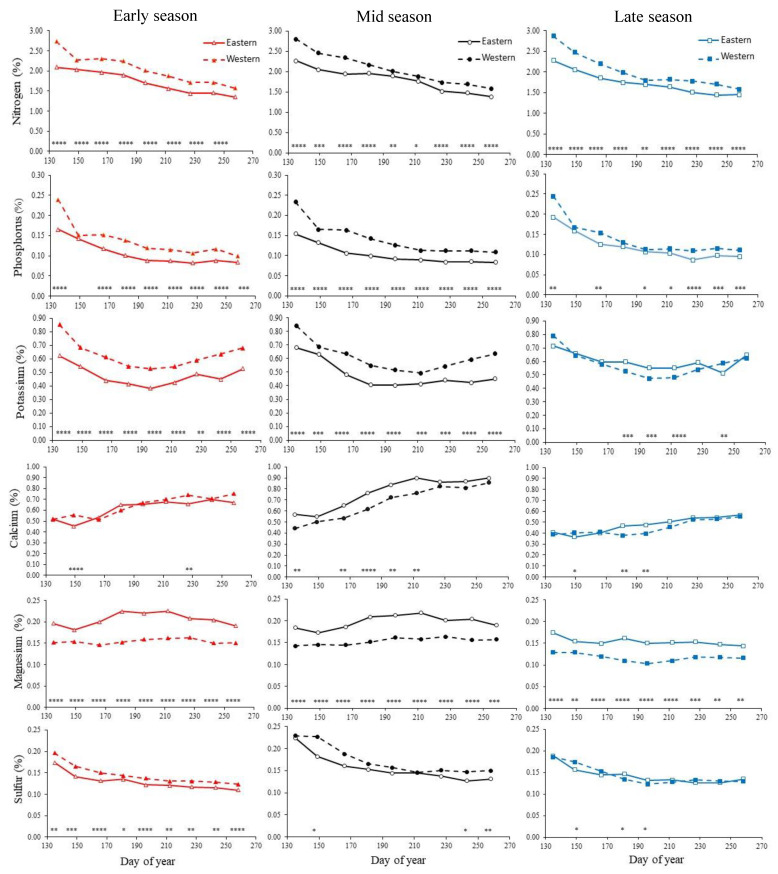
Concentrations of N, P, K, Ca, Mg, and S in recently expanded leaves of early-, mid-, and late-season cultivars of northern highbush blueberry in eastern and western Washington. Data are averaged over 3 years (2015–2017). ‘Duke’ was the early-season cultivar, ‘Draper’ the mid-season cultivar, and ‘Aurora’ and ‘Liberty’ the late-season cultivars in eastern Washington and western Washington, respectively. Each symbol represents the mean of the three field per cultivar in western Washington and four fields of ‘Duke’ and two fields each of ‘Draper’ and ‘Aurora’ in eastern Washington. Asterisks denote significance with: **** <0.0001, *** <0.001, ** <0.01, and * <0.05.

We also compared the nutrient concentrations between eastern and western Washington regions averaged across the three years in early, mid-, or late-season cultivars. Leaf N concentration declined slowly but consistently across the growing season and was always higher in western than in eastern Washington (Figure 1). At the onset of sample collection, concentrations of leaf tissue N averaged across three years were highest in late-season, intermediate in mid-season, and lowest in early-season cultivars. Leaf P also showed a decline with time and in all the three cultivars, leaves from western Washington exhibited higher P than eastern Washington on most days of sampling. Leaf tissue K concentrations declined until DOY 212 and then increased consistently after DOY 227 (Table 1). This increase was seen in all cultivars in both regions. Like N, P, and K, leaf S concentration decreased with time (Table 1). However, the average concentration was not statistically different after DOY 196 in late-season cultivars. Only ‘Duke’ differed throughout the growing season by region, with higher S in western than in eastern Washington (Figure 1). ‘Draper’ had higher leaf S concentrations in western than in eastern Washington on three sampling dates. The general trend of leaf Ca concentration was an increase with time that plateaued at DOY 227, averaged across the three years (Figure 1). The leaf Ca concentration was higher in eastern rather than in western Washington for the early and mid-season cultivars, but the late-season cultivar was higher in western than in eastern Washington at DOY 150 while opposite trends were observed on DOY 181 and DOY 196 (Figure 1). Leaf Mg concentration was highest in ‘Duke’, on average, and significantly lower for the mid-season cultivars followed by late-season cultivars (Table 1) for all three years. However, Mg concentration was sporadic with sampling time. There was a pronounced rise in Mg concentration at the mid-season time points in both growing regions for the early- and mid-season cultivars (Figure 1), although the magnitude was much higher in the eastern region than the western region.

Soil test results (Table 2) showed higher soil pH in eastern Washington than western Washington in all three years, while contrasting trends were observed in soil OM. Soil Ca and Mg in eastern Washington were also higher than in western Washington throughout the study. Soil K and Na were similarly higher in eastern Washington than western Washington for all three years. Time of sampling influenced soil pH, S, and Na in 2015; and soil S in 2017. In general, time of sampling did not influence the soil nutrient status. Cultivar influenced the soil nutrients except Ca, Mg, and K in 2015; S, Ca, and Mg in 2016 and Ca in 2017.

## 3. Discussion

Nutrient standards have been developed for northern highbush blueberry using information from various research trials and experiments. However, due to different growing conditions in eastern and western Washington, data collected from this study will be helpful in establishing macronutrient sufficiency standards, sampling guidelines, and nutrient requirements for blueberries east and west of the Cascade Range. Findings from this study can also be applied to other blueberry production regions with overlapping or similar pedo-climatic zones as the ones considered in our study. While four of our eastern Washington sites were grown organically and it is plausible organic management could impact soil and leaf nutrient concentrations, previous work conducted in a nearby state has shown that the overall patterns in blueberry leaf nutrient concentrations are similar between organic and conventional management [12] and both sufficiency standards and sampling guidelines do not need to differ based on organic management [1,4]. Therefore, organic management should not impact our key conclusions focused on optimal macronutrient sufficiency standards and sampling guidelines for the two contrasting growing regions.

It is important to note that the weather patterns across the three years were not similar and likely attributed to observed year effects. Overall, 2015 was the driest year of the study, with only 152 and 710 mm of precipitation in eastern and western Washington, respectively (Figure S1). In 2016, precipitation was intermediate with 188 and 904 mm in eastern and western Washington, respectively, compared to the wettest year, 2017, which received 195 and 997 mm. Similarly, 2015 was the warmest year of the study with average annual temperatures of 13.1 and 11.5 °C in eastern and western Washington, respectively. During the 2015 sampling period (DOY 130–260), average temperatures remained warm at 22.2 and 17.5 °C in eastern and western Washington, respectively. In both regions, the average annual temperature was slightly warmer in 2016 than 2017, at 12.3 and 10.9 °C in eastern and 11.1 and 10.0 °C in western Washington, respectively. However, the average temperature during the growing season (DOY 130–260) was slightly cooler in 2016 than 2017, with 20.3 and 21.3 °C in eastern and 16.3 and 16.6 °C in western Washington, respectively.

The leaf tissue nutrient concentrations between eastern and western regions indicate that most macronutrients had higher overall concentrations in western Washington, except for Ca and Mg, suggesting that a revised recommended set of ranges are needed for eastern production sites in the Pacific Northwest. This could plausibly be explained by higher soil OM content in soils from western Washington which helps in nutrient retention in soils [27,28]. Another explanation could be significantly lower pH in western Washington would have elevated the nutrient availability for blueberry plants in western Washington than eastern Washington. It is important to note that Ca and Mg concentrations were significantly lower in western Washington which could also be explained by lower pH in western Washington than eastern Washington [29,30].

Furthermore, our results suggest the time of year when leaf nutrient levels stabilize in eastern Washington was slightly later than in western Washington. Specifically, leaf N in eastern Washington, regardless of cultivar or region, decreased rapidly early in the growing season and began to level off between DOY 212 and 243. Our study indicates that when leaf N stabilizes in eastern Washington, a range of 1.25% to 1.75% N is recommended, compared to a range of 1.5% to 2.0% in western Washington (Table 3). Overall, leaf tissue P concentration declined until DOY 243 in both regions. At this time, a range of 0.08% to 0.15% P is recommended for eastern Washington, compared to 0.10% to 0.20% for western sites (Table 3). Leaf K concentration declined until DOY 212 then increased at DOY 227, at which point a leaf tissue range of 0.35% to 0.65% K is recommended for eastern Washington, compared to 0.40% to 0.70% in western sites, which is congruent with recommendations from Oregon [1] (Table 3). For leaf S, only ‘Duke’ differed throughout the growing season by region, with higher S in western than in eastern Washington (Figure 1). ‘Draper’ had higher leaf S concentrations in western than in eastern Washington on three sampling dates. However, the late-season cultivars were higher once in the western region and twice in the eastern region, which could be due to the differences in actual cultivars. Due to the small differences in leaf S between eastern and western Washington regions in mid- and late-seasons cultivars, a range of 0.10% to 0.20% S is recommended for both regions (Table 3).

Although K concentrations in leaf tissues were higher in western than in eastern Washington in all the three years, soil K was higher in eastern Washington than western Washington in 2015 and 2016, and no differences were observed in 2017. Higher overall precipitation in western Washington may have increased the availability of soil K for plant uptake and showed lower soil K values [31]. A similar explanation could also be true for other soil nutrients which were higher in eastern Washington than western Washington. Leaf tissue K concentrations declined until DOY 212 and then increased consistently after DOY 227, similar to the trend described by Bailey et al. [32] and contrary to the findings of Strik and Vance [12]. Although early and mid-season cultivars in western Washington had higher tissue K than the late-season cultivar, the opposite was found in eastern Washington from DOY 181 to 212 (Figure 1). Cultivar may account for the low tissue K concentrations found in ‘Liberty’ in western Washington, as Strik and Vance [12] described leaf samples collected from mid-July to autumn of ‘Liberty’ in western Oregon were among the lowest leaf K of six cultivars evaluated at conventional and organic sites. The leaf Ca concentration was higher in eastern rather than in western Washington for the early and mid-season cultivars, but the late-season cultivar was higher in western than in eastern Washington at DOY 150 (Figure 1). The general trend of leaf Ca concentration was an increase with time that plateaued at DOY 227. At this time, nutrient ranges were more variable than previous studies [12], thus a range of 0.40% to 1.00% Ca is recommended for both eastern and western Washington (Table 3). The concentration of Mg was sporadic with sampling time with a rise in concentration from DOY 150–190 in both growing regions for the early and mid-season cultivars. A range of 0.12% to 0.25% Mg is recommended in both eastern and western Washington growing regions, which is similar to what was recommended for western Oregon [1].

Overall, our results indicated that nutrient concentrations stabilized during later season sampling (DOY 212–243) periods in both Washington regions. Therefore, our findings lead to the recommendation of a slightly later sampling period compared to what is recommended in western Oregon studies, which recommend sampling from late July to mid-August [1,12]. This study also highlights the importance of developing sufficiency standards and sampling guidelines that consider regional environmental conditions under which the crop is grown. Findings from this study can be applied and used as a foundation for developing nutrient sufficiency and sampling guidelines for similar pedo-climactic zones around the world that cultivate soil-grown northern highbush blueberry.

## 4. Materials and Methods

*Sampling sites*. The study was conducted over three growing seasons (2015–2017) in commercial fields (6-year or older) of northern highbush blueberry in western and eastern Washington, USA. The sites included three fields each of ‘Duke’ (early), ‘Draper’(mid), and ‘Liberty’ (late) in Whatcom County and four fields of ‘Duke’ and two fields each of ‘Draper’ and ‘Aurora’ (late) in Benton County. Soil type varied among the sampling sites and ranged from mucks to sandy loams (Table 4). Air temperature (minimum, mean, and maximum) and precipitation data were collected daily at 15-minute intervals and downloaded from weather stations in Lynden (lat. 48°58’48’’ N, long. 122°25’48’’ W) and Prosser (lat. 46°15′0” N, long. 119°44’24’’ W) (weather.wsu.edu).

*Crop management*. Thirteen sites were managed using conventional practices and four were certified organic. All organic sites were in eastern Washington only. Soils in eastern Washington sites were acidified to suitable levels (4.5–5.5) using the grower standard practice of incorporating prilled S° prior to planting and irrigating the fields with acidified water. No acidification was necessary at the western Washington sites. All sites were irrigated by drip and fertilized following standard commercial industry practices for the region. Blueberry plants at all western sites were grown in raised beds mulched with Douglas fir [*Pseudotsuga menziesii* (Mirb.) Franco] sawdust. The sawdust was applied every 2–3 years and used for weed control and provision of organic matter accumulation. In eastern Washington, all eight sites except 4 and 8 had raised beds, sites 1, 2, 5, and 6 had raised beds with pre-plant incorporated orchard wood chips and a mulch of black weed mat (a woven polyethylene, polyester, and/or polypropylene geotextile), sites 3 and 7 had orchard wood chips with no weed mat, and sites 4 and 8 had mounded beds neither with no wood chips or weed mat. Orchard wood chips were sourced from adjacent apple and/or sweet cherry orchards and were aged prior to application.

*Cultivars*. The cultivars studied were selected to represent a range of ripening seasons and are commonly grown for commercial production in the PNW [33]. At all sites, ‘Duke’ and ‘Draper’ were chosen for early (May–June) and mid-fruiting (July), respectively. Due to regional differences the late fruiting cultivar, ‘Liberty’ (July–Auguest) was sampled exclusively in western Washington and ‘Aurora’ (August– September) was exclusively sampled in eastern Washington.

*Tissue sampling and analysis*. Leaf samples were collected nine times per year at each site and analyzed for nutrients. Three replicate areas were identified in the fields at each site, and within each replicate, a minimum of three rows were selected for sample collection. In all years, sampling was initiated on 14 to 18 May and occurred twice monthly on 28 May to 1 June, 11 to 15 June, 27 June to 1 July, 11 to 14 July, 26 July to 2 August, 10 to 18 August, 29 to 31 August, and 14 to 21 September. To group the samples due to slight differences between regions and years, they were assigned a day or year (DOY) designation that closely matched the 15th or 30th of each month which, in all cases, was within 4 days of actual sample collection. Tissue sampling consisted of collecting 75 of the most recent, fully expanded, disease-free whole leaves from lateral shoots at mid-canopy level in each of the three replications per standard recommendations [1]. Tissue was collected from both sides of the row.

Leaf samples were oven-dried at 60 °C for at least 48 h and ground to <0.42 mm with a Wiley mill (Thomas Scientific, Swedesboro, NJ, USA). The ground samples were then shipped to a commercial laboratory (Brookside Laboratories, New Bremen, OH, USA) for analysis of macronutrients, including N, P, K, Mg, Ca, and S. Micronutrients were analyzed and will be presented in complementary publications. Nitrogen was determined using a combustion analyzer with an induction furnace and a thermal conductivity detector, and the remaining nutrients were determined using an inductively coupled plasma spectrophotometer after wet ashing the samples in nitric/perchloric acid [34].

*Soil Sampling*. Soil samples were collected in the spring and fall of each year from the same general locations as the leaf samples. The samples were taken to a depth of 0.3 m using a 2.5-cm-diameter soil probe (AMS, American Falls, ID, USA). Fifteen samples were randomly collected from each replicate to form one composite sample. Depending on the year, samples were collected from 13 to 27 May and 24 August to 9 October. in eastern Washington and from 13 to 25 May and 11 to 15 September. in western Washington.

After sampling, soils from eastern Washington were air-dried under ambient laboratory conditions for a minimum of 3 days. Samples from western Washington were stored in the refrigerator at 1 °C until October and then sent to eastern Washington for drying. Some of the stored samples contained etiolated sprouts up to 15 cm in length. Dried soil was passed through a 2 mm sieve and sent to the same commercial laboratory as the leaf samples for analysis of total exchange capacity, CEC, pH, OM, S, Ca, Mg, K, H, and base saturation. Extractable soil S, Ca, Mg, and K were determined by ICP after extraction of the nutrients using the Mehlich 3 method [35]. Soil OM and pH were measured using Loss-On-Ignition at 360 °C [36] and the 1:1 soil:water method [37], respectively.

*Data analysis*. Statistical analyses were done using PROC GLIMMIX procedure in SAS v. 9.4 (SAS Institute, Cary, NC, USA). Due to significant year effects and interactions, analysis for variance (ANOVA) was conducted for each year separately for each parameter with region, day of year and cultivar as fixed effects. Data for all parameters were tested for normality using Shapiro–Wilk test. The mean separation was done LSD. Mean differences were considered significant at *p* ≤ 0.05.

## 5. Conclusions

The concentration of macronutrients in recently expanded leaves of northern highbush blueberry were affected by multiple interactions among year, region (western and eastern Washington), cultivar (early-, mid-, and late-season), and sampling date. Growing region effects showed tissue nutrient concentrations were overall higher in western Washington except for Ca and Mg with differences attributed to both climate and soil conditions, especially for Ca and Mg which are more abundant in eastern Washington soils. Periods of nutrient stabilization were later in both regions of Washington (1-31 Aug.) than in western Oregon, where many growers are referred to for tissue analysis and interpretation recommendations. Results from this study support later sampling periods for Washington growers (1–15 August) and a revised set of proposed ranges should be used for assessing plant nutrient status. These findings may be applicable to other blueberry production areas in the world that focus on soil-grown northern highbush cultivars with overlapping or similar pedo-climactic conditions and can be used as a foundation for further refinement. Growers should also continue to sample fields by cultivar which differed in leaf nutrient concentrations. More broadly, implications of this study underscore the importance of developing sufficiency standards and sampling guidelines tailored for a specific region under which northern highbush blueberry is grown.

## Figures and Tables

**Table 1:** The effect of growing region (R), cultivar (C), and day of year (DOY) on the concentration of tissue macronutrients in recently expanded leaves of early, mid-, and late-season cultivars of northern highbush blueberry in eastern and western Washington. ‘Duke’ was the early-season cultivar, ‘Draper’ the mid-season cultivar, and ‘Aurora’ and ‘Liberty’ the late-season cultivars in eastern Washington and western Washington, respectively. Non-significant interactions are highlighted with bold text.

		N		P		Mg		K		Ca		S	
		(%)											
2015													
Region (R)	East	1.67	b *	0.11	b	0.19	a	0.53	b	0.62	a	0.15	a
	West	2.1	a	0.15	a	0.14	b	0.58	a	0.57	b	0.14	b
Cultivar (C)	Early	1.83	b	0.13	a	0.18	a	0.54	b	0.6	b	0.14	b
	Mid	1.9	a	0.13	a	0.17	b	0.55	ab	0.7	a	0.15	a
	Late	1.9	a	0.14	a	0.14	c	0.58	a	0.5	c	0.14	b
Day of year (DOY)	135	2.62	a	0.23	a	0.16	bcde	0.82	a	0.46	d	0.21	a
	149	1.97	c	0.13	b	0.17	abcd	0.61	b	0.45	d	0.15	b
	166	2.12	b	0.14	b	0.16	cde	0.54	cd	0.47	d	0.14	b
	181	2.05	bc	0.13	b	0.18	a	0.51	ef	0.6	c	0.15	b
	196	1.73	d	0.11	cd	0.18	ab	0.46	f	0.66	ab	0.13	c
	212	1.67	d	0.1	d	0.18	ab	0.48	f	0.68	a	0.13	c
	227	1.64	de	0.11	cd	0.17	abc	0.53	de	0.66	ab	0.13	c
	243	1.56	e	0.12	cd	0.15	de	0.49	ef	0.62	bc	0.12	d
	258	1.45	f	0.11	cd	0.15	e	0.57	bc	0.66	ab	0.13	cd
	R	<0.0001		<0.0001		<0.0001		<0.0001		<0.0001		0.004	
	C	0.03		**0.09**		<0.0001		<0.0001		<0.0001		<0.0001	
	DOY	<0.0001		<0.0001		<0.0001		<0.0001		<0.0001		<0.0001	
	R × C	<0.0001		0.0002		<0.0001		<0.0001		0.0043		0.002	
	R × DOY	<0.0001		<0.0001		0.034		<0.0001		<0.0001		**0.1**	
	C × DOY	<0.0001		0.013		**0.08**		0.006		0.02		<0.0001	
	R × C × DOY	**0.22**		**0.39**		**0.92**		**0.43**		**0.16**		0.001	
2016													
Region (R)	East	1.73	b	0.1	b	0.19	a	0.5	b	0.66	a	0.13	b
	West	1.98	a	0.13	a	0.15	b	0.63	a	0.65	a	0.15	a
Cultivar (C)	Early	1.85	b	0.1	b	0.19	a	0.56	b	0.69	b	0.13	b
	Mid	1.9	a	0.12	a	0.17	b	0.55	b	0.8	a	0.16	a
	Late	1.82	c	0.12	a	0.13	c	0.6	a	0.48	c	0.13	b
Day of year (DOY)	135	2.36	a	0.17	a	0.18	a	0.7	a	0.52	fg	0.17	a
	149	2.03	bc	0.14	b	0.15	b	0.62	bc	0.48	g	0.15	b
	166	2.07	b	0.12	c	0.17	ab	0.52	e	0.58	ef	0.15	b
	181	1.98	c	0.11	d	0.16	ab	0.48	e	0.62	de	0.13	c
	196	1.88	d	0.1	e	0.17	ab	0.48	e	0.67	cd	0.13	c
	212	1.72	e	0.1	ef	0.17	a	0.49	e	0.72	bc	0.13	c
	227	1.59	f	0.09	fg	0.17	a	0.58	cd	0.77	ab	0.13	c
	243	1.59	f	0.09	fg	0.17	a	0.56	d	0.77	ab	0.13	c
	258	1.5	g	0.09	g	0.17	a	0.65	b	0.81	a	0.13	c
	R	<0.0001		<0.0001		<0.0001		0.0003		**0.91**		<0.0001	
	C	0.0007		0.0002		<0.0001		<0.0001		<0.0001		<0.0001	
	DOY	<0.0001		<0.0001		0.01		<0.0001		<0.0001		<0.0001	
	R × C	**0.053**		<0.0001		0.02		<0.0001		<0.0001		0.0002	
	R × DOY	0.009		0.002		0.009		<0.0001		0.001		<0.0001	
	C × DOY	<0.0001		0.0004		0.0001		0.030		<0.0001		0.02	
	R × C × DOY	0.009		<0.0001		**0.86**		**0.49**		**0.38**		**0.87**	
2017													
Region (R)	East	1.83	b	0.11	b	0.19	a	0.5	b	0.57	a	0.14	b
	West	2	a	0.13	a	0.14	b	0.58	a	0.55	a	0.16	a
Cultivar (C)	Early	1.86	b	0.11	b	0.18	a	0.51	c	0.59	b	0.13	c
	Mid	2	a	0.12	ab	0.16	b	0.54	b	0.68	a	0.18	a
	Late	1.9	b	0.13	a	0.13	c	0.57	a	0.42	c	0.15	b
Day of year (DOY)	135	2.27	b	0.19	a	0.16	a	0.61	ab	0.42	f	0.21	a
	149	2.47	a	0.17	b	0.16	a	0.64	a	0.47	ef	0.19	a
	166	2.15	c	0.15	c	0.156	a	0.59	b	0.47	ef	0.17	b
	181	1.99	d	0.12	d	0.161	a	0.51	d	0.52	de	0.15	c
	196	1.93	de	0.11	e	0.162	a	0.47	e	0.54	cd	0.14	cd
	212	1.86	e	0.11	e	0.167	a	0.46	e	0.59	bc	0.14	cd
	227	1.63	f	0.09	f	0.164	a	0.49	de	0.63	ab	0.13	d
	243	1.59	f	0.1	f	0.169	a	0.55	c	0.69	a	0.13	d
	258	1.52	g	0.1	f	0.161	a	0.56	c	0.66	a	0.13	d
	R	<0.0001		<0.0001		<0.0001		<0.0001		**0.051**		<0.0001	
	C	<0.0001		<0.0001		<0.0001		<0.0001		<0.0001		<0.0001	
	DOY	<0.0001		<0.0001		**0.17**		<0.0001		<0.0001		<0.0001	
	R × C	**0.47**		<0.0001		0.0042		<0.0001		<0.0001		<0.0001	
	R × DOY	<0.0001		<0.0001		<0.0001		**0.1145**		0.001		0.0016	
	C × DOY	<0.0001		<0.0001		<0.0001		<0.0001		<0.0001		0.0001	
	R × C × DOY	<0.0001		<0.0001		**0.68**		**0.214**		**0.7209**		**0.2307**	

* Means with the same letter within a column for region, cultivar and DOY are not significantly different at α = 0.05. Bolded *P*-values indicate a lack of statistical significance.

**Table 2:** The effect of growing region (R), cultivar (C), and day of year (DOY) on the soil nutrients in northern highbush blueberry systems in eastern and western Washington. ‘Duke’ was the early-season cultivar, ‘Draper’ the mid-season cultivar, and ‘Aurora’ and ‘Liberty’ the late-season cultivars in eastern Washington and western Washington, respectively. Non-significant effects are highlighted with bold text.

		CEC		pH		OM		S		Ca		Mg		K		Na	
		meq/100 g soil				(%)		--------------------------------------(ppm)------------------------------------									
2015																	
Region (R)	East	22.7	a *	4.9	a	2.9	b	90.6	a	1304.5	a	305.5	a	229.3	a	111.7	a
	West	17.1	b	4.4	b	18.9	a	112.6	a	870.6	b	135.1	b	178.6	b	31.9	b
Cultivar (C)	Early	18.8	b	4.7	ab	9.9	ab	109.6	a	1068.8	a	206.7	a	197.9	a	72.3	b
	Mid	23.4	a	4.5	b	15.3	a	121.6	a	1117.2	a	213.5	a	191.7	a	99.1	a
	Late	17.4	b	4.9	a	7.5	b	73.6	b	1076.6	a	240.8	a	222.2	a	44.2	c
Sampling time (T)	May	20.5	a	4.5	b	10.9	a	91.4	a	1010.4	a	191.9	b	188.3	b	45.9	b
	Sep	19.2	a	4.9	a	10.8	a	111.8	a	1164.7	a	248.8	a	219.6	a	97.8	a
	R	0.002		0.0002		<0.0001		**0.1405**		0.0051		0.001		0.0004		<0.0001	
	C	0.0226		0.0157		0.0364		0.0347		**0.9527**		**0.4134**		**0.1899**		0.0002	
	T	**0.476**		<0.0001		**0.9711**		**0.1692**		**0.2197**		0.0117		0.0251		<0.0001	
	R × C	**0.1786**		0.0246		**0.0563**		<0.0001		**0.5242**		0.0441		**0.1152**		0.0011	
	R × T	**0.1476**		**0.0715**		**0.9983**		**0.2765**		0.0278		0.0259		**0.0112**		0.0001	
	C × T	**0.3342**		**0.9177**		**0.9589**		**0.936**		**0.4583**		**0.2965**		**0.6171**		0.0137	
	R × C × T	**0.9355**		**0.8863**		**0.973**		**0.627**		**0.8419**		**0.7855**		**0.4498**		0.0082	
2016																	
Region (R)	East	17.7	a	5.2	a	3.1	b	52.4	b	1198.8	a	285.2	a	223.9	a	91.4	a
	West	16.9	a	4.8	b	17.9	a	108.2	a	1067.5	a	137.7	b	189.1	b	40.6	b
Cultivar (C)	Early	13.9	b	5.1	a	7.9	b	80.8	a	1000.4	a	191.9	a	206.1	ab	58.4	b
	Mid	21.6	a	4.8	b	16.5	a	79.3	a	1250.9	a	217.7	a	174.4	b	98.5	a
	Late	16.5	b	5.1	a	6.9	b	80.8	a	1148.2	a	224.7	a	238.9	a	41.1	c
Sampling time (T)	May	17.7	a	4.9	a	10.8	a	77.5	a	1108.2	a	200.4	a	213.2	a	65.1	a
	Sep	16.9	a	5.1	a	10.1	a	83.1	a	1158.2	a	222.5	a	199.8	a	66.9	a
	R	**0.5606**		<0.0001		<0.0001		<0.0001		**0.2031**		<0.0001		0.0425		<0.0001	
	C	<0.0001		0.0015		0.001		**0.9918**		**0.1177**		**0.0934**		0.016		<0.0001	
	T	**0.5704**		**0.1593**		**0.7191**		**0.6147**		**0.6269**		**0.1027**		**0.4352**		**0.7689**	
	R × C	**0.1068**		**0.0766**		0.0015		0.0007		0.0177		0.0029		**0.5455**		<0.0001	
	R × T	**0.5278**		**0.3561**		**0.8454**		**0.4935**		**0.424**		**0.1614**		**0.356**		0.009	
	C × T	**0.6096**		**0.2063**		**0.6663**		**0.287**		**0.8404**		**0.4854**		0.0309		0.0377	
	R × C × T	**0.8725**		**0.2659**		**0.605**		**0.895**		**0.8594**		**0.7291**		**0.5473**		**0.1652**	
2017																	
Region (R)	East	16.9	b	5.4	a	2.5	b	43.2	b	1275.2	a	305.4	a	215.1	a	72.9	a
	West	20.1	a	4.4	b	17.6	a	131.1	a	1027.5	b	168.9	b	242.5	a	37.1	b
Cultivar (C)	Early	16.5	b	4.9	a	7.6	b	108.9	a	1118.4	a	212.8	b	227.4	b	56.3	a
	Mid	21.1	a	4.7	b	16.4	a	69.9	b	1215.4	a	226.3	ab	179.6	b	66.4	a
	Late	18.2	ab	5.1	a	6.2	b	82.4	ab	1120.2	a	272.4	a	279.4	a	42.4	b
Sampling time (T)	May	18.5	a	4.8	a	10.3	a	73.9	b	1093.6	a	232.3	a	238.9	a	52.2	a
	Sep	18.6	a	4.9	a	9.9	a	100.2	a	1209.1	a	242.0	a	218.8	a	57.9	a
	R	0.0103		<0.0001		<0.0001		<0.0001		0.0276		<0.0001		**0.2001**		<0.0001	
	C	0.0079		0.0198		0.0007		0.0362		**0.7241**		0.0449		0.0021		0.0014	
	T	**0.9212**		**0.1757**		**0.8529**		0.0469		**0.299**		**0.6299**		**0.3444**		**0.2502**	
	R × C	0.0494		0.003		0.0013		0.0029		**0.0641**		0.0112		**0.2705**		0.0029	
	R × T	**0.6643**		**0.1217**		**0.7699**		**0.3347**		**0.6621**		**0.9608**		**0.3192**		0.003	
	C × T	**0.7728**		**0.6798**		**0.6555**		**0.797**		**0.6875**		**0.5377**		**0.1162**		**0.5641**	
	R × C × T	**0.7372**		**0.7182**		**0.7084**		**0.8179**		**0.9944**		**0.6428**		**0.2011**		**0.9523**	

* Means with the same letter within a column for region, cultivar and DOY are not significantly different at α = 0.05. Bolded *P*-values indicate a lack of statistical significance.

**Table 3:** Recommended ranges of northern highbush blueberry tissue macronutrient levels in eastern and western Washington when leaf samples are collected between 1–31 August. (DOY 212–243). Recommended ranges are listed from previous research in western Oregon [1] and Michigan [17].

		Washington		Michigan	Western Oregon
Element	Unit	Eastern	Western		
Nitrogen (N)	%	1.25–1.75	1.5–2.0	1.70–2.10	1.76–2.0
Phosphorus (P)	%	0.08–0.15	0.10–0.20	0.08–0.40	0.10–0.40
Potassium (K)	%	0.35–0.65	0.40–0.70	0.40–0.65	0.41–0.70
Calcium (Ca)	%	0.40–1.00		0.30–0.80	0.41–0.80
Magnesium (Mg)	%	0.12–0.25		0.15–0.30	0.13–0.25
Sulfur (S)	%	0.10–0.20		0.12–0.20	0.11–0.16

**Table 4:** Description of cultivars and soil type characteristics for sampled study sites in eastern and western Washington, USA.

Sampling Site	Cultivar	Soil Type
Eastern Washington		
1	‘Duke’	Prosser—coarse-loamy, mixed, superactive, mesic Xeric Haplocambid
		Warden—coarse-silty, mixed, superactive, mesic Xeric Haplocambid
2	‘Duke’	Warden- coarse-silty, mixed, superactive, mesic Xeric Haplocambid
3	‘Duke’	Warden—coarse-silty, mixed, superactive, mesic Xeric Haplocambid
4	‘Duke’	Prosser—coarse-loamy, mixed, superactive, mesic Xeric Haplocambid
		Warden—coarse-silty, mixed, superactive, mesic Xeric Haplocambid
5	‘Draper’	Prosser—coarse-loamy, mixed, superactive, mesic Xeric Haplocambid
		Warden—coarse-silty, mixed, superactive, mesic Xeric Haplocambid
6	‘Draper’	Scooteney—loamy, mixed, superactive, mesic Lithic Xeric Haplocambid
		Starbuck—coarse-loamy, mixed, superactive, mesic Xeric Haplocambid
7	‘Aurora’	Warden—coarse-silty, mixed, superactive, mesic Xeric Haplocambid
8	‘Aurora’	Prosser—coarse-loamy, mixed, superactive, mesic Xeric Haplocambid
		Warden—coarse-silty, mixed, superactive, mesic Xeric Haplocambid
Western Washington		
1	‘Duke’	Pangborn muck, drained, Dysic, mesic Typic Haplosaprists
2	‘Duke’	Tromp loam, Sandy, mixed, mesic Aquic Haplorthods
3	‘Duke’	Tromp loam, Sandy, mixed, mesic Aquic Haplorthods
4	‘Draper’	Pangborn muck, drained, Dysic, mesic Typic Haplosaprists
5	‘Draper’	Labounty silt loam, drained, Fine-loamy, isotic, mesic Aquandic Endoaqualfs
		Shalcar muck, drained, Loamy, mixed, euic, mesic Terric Haplosaprists
6	‘Draper’	Edmonds-Woodlyn loams, mesic, ortstein Andic Endoaquods
7	‘Liberty’	Labounty silt loam, drained, Fine-loamy, isotic, mesic Aquandic Endoaqualfs
		Shalcar muck, drained, Loamy, mixed, euic, mesic Terric Haplosaprists
8	‘Liberty’	Edmonds-Woodlyn loams, mesic, ortstein Andic Endoaquods
9	‘Liberty’	Puyallup fine sandy loam, isotic over mixed, mesic Fluventic Haploxerolls

## Data Availability

All data included in the main text and supplementary materials.

## Supplementary Materials

The following supporting information can be downloaded at: https://www.mdpi.com/article/10.3390/plants11233376/s1, Figure S1: Annual mean temperature and cumulative precipitation from 2015–2017 in eastern and western Washington. In both regions, leaves were collected for nutrient analysis from commercial fields of northern highbush blueberry on day 135–258 (are between the red vertical lines).
