# Supplementary material for: Leaf Tissue Macronutrient Standards for Northern Highbush Blueberry Grown in Contrasting Environments
Source: Plants (Basel). 2022 Dec 5;11(23):3376. doi: 10.3390/plants11233376 (PMC9735984; doi:10.3390/plants11233376)
Supplement: Supplementary file 1 [file plants-11-03376-s001.zip › plants-2031119-supplementary.pdf]

Supplementary File

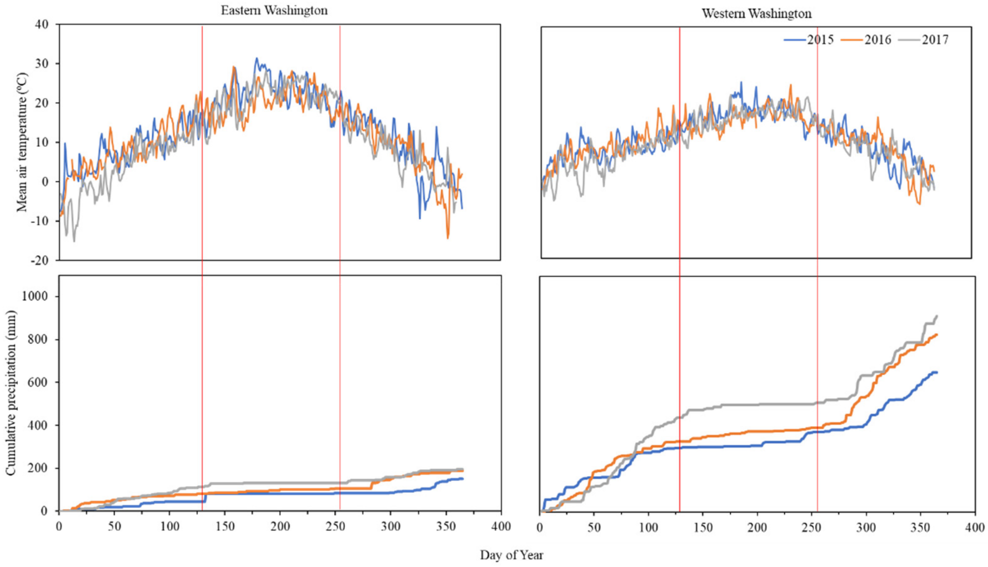

Supplemental Figure S1. Annual mean temperature and cumulative precipitation from 2015 – 2017 in eastern and western Washington. In both regions, leaves were collected for nutrient analysis from commercial fields of northern highbush blueberry on day 135 – 258 (are between the red vertical lines).
